# Interrater and intra-rater reliability of the VERA-2R tool

**DOI:** 10.3389/fpsyt.2023.1236295

**Published:** 2023-09-27

**Authors:** Nils Duits, Maaike Kempes

**Affiliations:** Netherlands Institute of Forensic Psychiatry and Psychology (NIFP), Utrecht, Netherlands

**Keywords:** terrorism, violent extremism, risk assessment, interrater reliability, intra-rater reliability, VERA-2R

## Abstract

**Introduction:**

The Violent Extremism Risk Assessment - Version 2 Revised (VERA-2R) is an evidence-based structured professional judgement (SPJ) tool for ideologically motivated violence. Use of the tool can help professionals in risk assessment and risk management of suspected and convicted terrorists and violent extremists at different stages within the criminal process. It is important that the tool leads to reliable and valid risk assessments.

**Methods:**

The present study aimed to establish the reliability of the VERA-2R, focusing on the interrater- and intrarater reliability. Trained researchers assessed the interrater- and intrarater reliability, respectively in 30 and 33 cases of Dutch convicted terrorist offenders, on the basis of extensive judicial files.

**Results:**

In general, the average amount of agreement on the indicators and structured risk judgements can be classified as good to excellent, for both the interrater- and intra-rater reliability. However, six indicators were found to have low reliability. Above clarifying the reliability of the VERA-2R, this study also showed how the interrater- and intrarater reliability of a SPJ tool can be investigated with trained assessors based on judicial files. This can be of added value, because existing reliability studies often use case vignettes, have a small sample size and/or do not include a stringent training program.

**Discussion:**

With this research, we can make more substantiated recommendations on how the VERA-2R can be improved. This could lead to more accurate risk assessments and risk management strategies. However, in order to develop a more reliable and valid VERA-2R instrument, the remaining psychometric properties must also be investigated and published. That will be discussed in a future article.

## Introduction

Although many definitions and types of terrorism exist, one can define terrorism as ‘ideologically inspired (preparations for) the perpetration of acts of violence against human life or of acts that cause society-disrupting damage, with the aim of creating a climate of serious fear among (part of) the general population, bring about social change and/or influence political decision-making’ (1–3). Therefore, being a member of or participation in a terrorist organization, threatening with terrorist attacks, recruiting, and financing of terrorism are also considered to be terrorist offenses. Violent extremism can be described as the beliefs and actions of individuals who support or use violence to achieve ideological, religious or political goals (4).

Violent extremism and terrorism can have a wide range of detrimental consequences for society, including, among other things, the loss of human life, material damage, emotional impact, not to mention the damage to the democratic process and prevailing legal order. Individuals who are imprisoned for a violent extremist or terrorist offence, as well as prisoners who are radicalised in prison, pose a serious security threat, both during their imprisonment and after their release from prison (5). The average prison sentence for terrorist offences in the reported proceedings in European Member States in 2021 was six years (5). Tackling potentially violent, extremist lone actors and ensuring safe reintegration measures for persons who have been convicted of terrorist offences, both during and after detention will require special attention. This is all the more important because individuals who are convicted of terrorist offences will regularly be released from detention in the years ahead (1). Therefore, a more evidence-based professional approach to violent extremism and terrorism risk assessment and risk management is urgently needed (6). Determining the psychometric properties of the currently available risk assessment tools for ideologically motivated violence is thus of importance. In the present study, we seek to obtain greater insight into the reliability of the Violent Extremism Risk Assessment - Version 2 Revised (VERA-2R), focussing on the interrater- and intra-rater reliability.

### Violent extremism and terrorism risk assessment and VERA-2R

Evidence-based violence risk assessment can be defined as the process of collecting information about individuals, in a way that adheres to, and is guided by, the available scientific and professional knowledge-base, both for the purposes of understanding whether individuals constitute a risk of engaging in violent behaviour in the near future and determining which subsequent actions should be taken to prevent this violence from occurring (7). Evidence-based risk assessments can inform risk management strategies and interventions by identifying the possible risk scenarios (8). Furthermore, they can help to ensure transparent decision-making, avoid recurring decision-making errors, and enhance the level of understanding both within and across multidisciplinary teams (9, 10). Traditionally, researchers and practitioners have distinguished between three generations of risk assessment methods: (1) unstructured clinical judgements, which are risk assessments that are based solely on clinicians’ experience and knowledge (11); (2) the actuarial method, which involves using a fixed algorithm of group-based estimates to combine evidence-based indicators into a final risk judgement (8); (3) Structured Professional Judgement (SPJ), which combines evidence-based knowledge about risk analysis and the principle of structured professional judgement (8, 12).

In order to arrive at a structured final risk judgement, assessors must use their professional judgement to integrate, combine and weigh all the relevant information and data related to the evidence-based indicators [(13–15), Logan, 2014]. Scientific experts consider SPJ to be the most suitable method for assessing the risk of ideologically motivated individuals (14, 16, 17). Given that prior analyses have demonstrated that most of the currently available risk assessment instruments for general violence are not relevant to the idiosyncrasies of terrorists and violent extremists, the need for a specialized SPJ instrument for ideologically motivated violence emerged (17). This subsequently led to the development of different risk assessment tools for violent extremism and terrorism (14, 18, 19).

The VERA was the first specialized tool for conducting individual risk assessments for terrorists and violent extremists (17, 20). In response to feedback from terrorism experts, the VERA was subsequently revised and renamed the Violent Extremism Risk Assessment - Version 2 (VERA-2) (20, 21). In 2018, the most recent version of the instrument, identified as the Violent Extremism Risk Assessment - Version 2 Revised (VERA-2R), became available. This version incorporated several further revisions and improvements based on additional research into the indicators associated with violent extremism and terrorism (20, 22).

The VERA-2R can be used to establish the risk status for individuals who have been accused, arrested or convicted of a violent extremist or terrorist offence (20). Adhering to the SPJ methodology, the VERA-2R acknowledges that the weighting of the indicators should not be defined beforehand, due to the fact that the relevance of the indicators may vary depending on the specific context of the individual (20). Therefore, professional judgement must be exercised to integrate, combine and weigh all the relevant information and data related to the relevant indicators to develop a meaningful appreciation for the risk propensity of the individual (13, 20). Based on the resulting structured final risk judgement, different risk scenarios must be formulated along with a corresponding risk management strategy for each of these scenarios (12, 15).

The VERA-2R is widely used by trained professionals, both within and outside Europe, to assist with decision-making within various stages of the criminal justice process (23). In pre-trial settings, the VERA-2R is used by probation officers, forensic psychiatrists and forensic psychologists to improve the risk recommendations they proffer to the court (23, 24). In post-trial settings, the VERA-2R facilitates a risk analyses that (a) forms the bases for a tailor-made approach and differentiated placement policy, (b) supports decisions about the continuation of intervention and/or rehabilitation programs, (c) helps to determine whether prisoners are able to be released on parole, and (d) is used to establish the risk that the persons under supervision will commit a violent extremist or terrorist offence in the future (20, 23, 24).

### Psychometric properties and the VERA-2R

Risk assessments play a significant role in terms of combatting violent extremism and terrorism (6, 25). Therefore, it is of importance that the risk assessment tools for ideologically motivated violence provide reliable and valid risk estimates. Reliability pertains to the extent to which a measurement is stable, consistent, predictable, accurate and free from random error (26). Validity concerns the extent to which an instrument measures what it purports to measure (27).

Despite the importance of validation, due to the relative dearth of thorough scientific research, there remains scarce knowledge about the reliability and validity of risk assessment instruments (28). With respect to the VERA-2R, professionals have reached consensus on the face validity and content validity (20). Face validity refers to the degree to which an instrument creates the impression that it encompasses the entirety of the concept that it claims to measure (29), while content validity can be defined as the degree to which an instrument adequately represents all the relevant facets of a given construct (30).

Interrater reliability pertains to the degree to which two or more observers independently score the same ratings for the feature that is being observed or measured (31). High interrater reliability is of importance, since structured risk judgements serve as the basis for important decisions in the criminal justice process (19), and therefore should be wholly independent of the observers or professional assessors (32).

Although a previous study has demonstrated a high interrater reliability for the VERA (33), this result cannot be generalized to the VERA-2R, because the VERA-2R incorporates revisions that can impact upon the interrater reliability. Next to this, Beardsley and Beech (33) also used case vignettes, had a small sample size and did not include a stringent training program, which, in turn, may have impacted upon their findings. In light of the above, further research is needed to establish the interrater reliability of the VERA-2R.

In addition to this, previous research has not established the intra-rater reliability of the tool yet. Intra-rater reliability refers to “the extent to which an assessor, reusing the same instrument, consistently assigns the same ratings over time while examining a single set of data” (Belur et al., 2021). A high level of intra-rater reliability is a prerequisite for risk assessment instruments, insofar as it indicates that the tool can measure a constant phenomenon in the same way over time (Hopkins, 2000). Better reproducibility suggests better precision of single measurements, which is a requirement for better tracking of changes in measurements in research or practice settings (Hopkins, 2000). This, subsequently, provides certainty that the tool can be used for repeated measurements. The dynamic nature of the process of radicalization to ideologically motivated violence and vice versa (20), in combination with the fact that, as a response to this, repeated measurements with the VERA-2R are necessary and have to be implemented in the justice system, makes this of utmost importance (23).

The term intra-rater reliability is sometimes wrongly used interchangeably with the term test–retest reliability (Holmefur et al., 2009). However, there is a significant difference between the two. While intra-rater reliability refers to the agreement between repeated observations of the same test session, test–retest reliability includes two different test sessions. Test–retest reliability therefore inevitably includes intra-rater error (Holmefur et al., 2009). Since our research design includes extensive, static judicial files which will be examined twice by the same assessor, in this study we will focus on the intra-rater reliability.

### Interrater and intra-rater reliability of the VERA-2R

This study constitutes the first part of an extensive validation project and seeks to provide insight into the interrater and intra-rater reliability of the VERA-2R. Given that a prior study produced promising results regarding the interrater reliability of the VERA, we hypothesize that the VERA-2R will also have high interrater reliability. Furthermore, since the study of Beardsley and Beech (33) provided a first indication that the VERA incorporates clearly expressed items and encoding rules, and we expect that this will also have a positive effect on the intra-rater reliability, we hypothesize to find high intra-rater reliability.

## Methods interrater reliability: assessors and cases

The assessors included in this study were two Dutch researchers (one male, one female) with a bachelor and master degree in psychology and/or criminology. At the time of the study, the researchers were employed by the Netherlands Institute of Forensic Psychiatry and Psychology (NIFP) of the Dutch Ministry of Justice and Security. The assessors took part in a professional two-day training course to obtain an in-depth understanding of the instrument and to acquire experience in applying the VERA-2R indicators and forming structured risk judgements.

To assess the interrater reliability of the VERA-2R, both assessors independently rated a sample of 30 terrorist cases based on extensive judicial files. These files were provided by the Dutch Public Prosecution Service and included a mental health assessment, a probation report, a transcript of the verdict, a police report, a criminal record and/or information from intelligence services.

The sample comprised 24 men and 6 women who were convicted of terrorist offences in the Netherlands between 2012 and 2019. The subjects’ ages ranged from 15–47 at the time of their terrorist act (*M*_age_ = 25.47, *SD* = 7.99). Ninety percent of the subjects were from a migrant background, 3.3% of the subjects had no migration background, and for 6.7% the background was unknown. Most of the subjects from a migrant background had parents who were born in Morocco or were born there themselves (48.1%), followed by persons with a Turkish background (18.5%). Consequently, the sample appears to be representative of the target population with respect to age, gender and migration background, since similar descriptive statistics were found in previous studies on Dutch jihadists (34–37).

### Materials: VERA-2R

The VERA-2R contains 34 risk and protective indicators specifically related to the risk of violent extremism and terrorism (20). The VERA-2R indicators are divided into five domains: Beliefs, Attitudes and Ideology (BA), Social Context and Intention (SCI), History, Action and Capacity (HAC), Commitment and Motivation (CM), and Protective and risk-mitigating indicators (P). The scientific basis for each indicator is explained, along with the underlying criteria for the three rating levels: low, moderate or high. A risk indicator is rated as ‘low’ if the risk-promoting indicator characteristics are objectively not present, as ‘moderate’ if the risk-promoting indicator characteristics are present to a specified level, and as ‘high’ if the risk-promoting indicator characteristics are clearly present or present to a high level. The protective indicators are scored in reverse, which is to say that lower scores indicate a higher level of risk (20). A protective indicator is rated as ‘low’ if no risk-mitigating indicator characteristics are present, as ‘moderate’ if some risk-mitigating indicator characteristics are present, and as ‘high’ if clear risk-mitigating indicator characteristics are present (20). It is important to stress here that the VERA-2R does not provide a numerical score for the ratings (20); however, for the purposes of this study, the numerical scores ‘0’, ‘1’ and ‘2’ are assigned to the ratings ‘low’, ‘moderate’ and ‘high’, respectively. Based on the assumption that the indicator characteristics will be cited in the judicial file if they are present, we decided to assign the numerical score ‘0’ if the judicial file did not contain information about an indicator.

The VERA-2R also includes 11 additional indicators, which may contribute to a person’s vulnerability to engage in future acts of violent extremism and terrorism, when combined with the presence of ideological, contextual, and motivational indicators identified in the VERA-2R (20). These additional indicators are divided into three domains: Criminal History (CH), Personal History (PH) and Mental Disorder (MD). The scientific basis for each indicator is explained, along with the criteria for the two rating levels: not present or present. The rating ‘not present’ (0) corresponds to the absence of the additional indicator characteristics, while the rating ‘present’ (1) corresponds to the presence of the additional indicator characteristics (20). If a judicial file did not contain information about an indicator, then the numerical score ‘0’ was assigned.

After carefully considering the indicators, the assessor then assigns structured risk judgements to the VERA-2R domains (20). Subsequently, a structured final risk judgment is made in terms of the likelihood of an individual engaging in ideologically motivated violence. The structured risk judgements are formulated in a risk narrative, as well as rated on a scale of low (0), moderate (1), and high (2) (20). Furthermore, different risk scenarios are identified with a risk management strategy for each of these scenarios (12, 15).

#### Research design

Given that our research design included trained researchers and extensive judicial files, the risk assessments conducted as part of this study closely resemble VERA-2R assessments in practice. However, we must acknowledge that, in practice, VERA-2R assessments are carried out by professionals with extensive experience in carrying out individual risk assessments and preferably also include information obtained from a direct interview with the person concerned. Therefore, while the research design can be said to reflect the practice of structured professional risk assessment as closely as possible, it must be defined as a research setting.

In the research design, we also took into account the fact that the information upon which this study is based originates from comprehensive judicial files, which include information from a range of different sources, such as the police, the Public Prosecution Service, and forensic psychiatrists and psychologists. In order to ensure that the assessors did not assign different ratings to the indicators, as a result of relying on different sources that may have differing opinions about whether (and to what extent) the indicator characteristics are present, we decided to inform the second assessor which source the first assessor had used.

#### Security and privacy

To ensure that there were no risks to the privacy of the subjects included in this study, we anonymized the data. Moreover, with regard to data protection, we stored the anonymized dataset in a secure digital environment, in order to protect the information against misuse, unauthorized access, disclosure and theft.

### Statistical analysis

The statistical analyses in this study were carried out using IBM SPSS Statistics for Mac Version 25.0. The interrater reliability of the VERA-2R was examined by means of the intraclass correlation coefficient (ICC), using the two-way random effects model and absolute agreement type (38). The intraclass correlation coefficient (ICC) was chosen as the reliability index, because extant literature has showed that ICC is one of the most commonly-used statistics for interval variables (38). The ICC values were established for both the VERA-2R indicators and structured risk judgements. Furthermore, mean ICC values were determined by calculating the average amount of agreement over the VERA-2R indicators. Interpretation of ICCs were based on the critical values for single measures provided by Fleiss (39): ICC < 0.40 = poor, 0.40 ≤ ICC < 0.60 = fair, 0.60 ≤ ICC < 0.75 = good and ICC ≥ 0.75 = excellent.

The interrater reliability of the additional indicators was examined by means of Cohen’s kappa (κ). Furthermore, mean kappa values were determined by calculating the average amount of agreement over the additional indicators. Cohen’s kappa (κ) was chosen as the reliability index for the additional indicators, because extant literature has showed that Cohen’s kappa is one of the most commonly-used statistics for nominal variables (38). The kappa values were interpreted in accordance with the guidelines outlined by Landis and Koch (40): κ ≤ 0.20 = slight, 0.20 < κ ≤ 0.40 = fair, 0.40 < κ ≤ 0.60 = moderate, 0.60 < κ ≤ 0.80 = good and 0.80 < κ ≤ 0.1.00 = excellent.

### Results interrater reliability VERA-2R indicators

Table 1 shows the ICCs for the VERA-2R indicators. The indicators within the ‘Beliefs, Attitudes and Ideology’ domain (BA) all have good to excellent interrater reliability, with ICC values ranging from 0.64 (indicator BA.4) to 0.90 (indicator BA.6). The mean ICC value of the indicators is 0.79, which indicates excellent interrater reliability.

**Table 1:** Interrater reliability VERA-2R.

Domain and indicator	*N*	ICC	95% CI
**BA. Beliefs, Attitudes and Ideology**			
BA.1 Commitment to ideology that justifies violence	30	0.72***	0.49–0.85
BA.2 Perceived grievances and/or perceived injustice	30	0.81***	0.64–0.91
BA.3 Dehumanization of designated targets associated with injustice	30	0.72***	0.49–0.86
BA.4 Rejection of democratic society and values	30	0.64***	0.37–0.81
BA.5 Expressed emotions in response to perceived injustice	30	0.89***	0.79–0.95
BA.6 Hostility to national identity	30	0.90***	0.80–0.95
BA.7 Lack of empathy and understanding for those outside one’s own group	30	85***	0.71–0.93
Mean domain BA		0.79	

**SCI. Social Context and Intention**			
SCI.1 Seeker, user or developer of violent extremist materials	30	0.97***	0.94–0.99
SCI.2 Target for attack identified (person, group, location)	30	0.87***	0.74–0.93
SCI.3 Personal contact with violent extremists (informal or social context)	30	0.72***	0.48–0.86
SCI.4 Expressed intention to commit acts of violent extremism	30	0.83***	0.66–0.92
SCI.5 Expressed willingness and/or preparation to die for a cause or belief	30	0.83***	0.67–0.91
SCI.6 Planning, preparation of acts of violent extremism	30	0.53**	0.22–0.74
SCI.7 Susceptibility to influence, control or indoctrination	30	0.98***	0.95–0.99
Mean domain SCI		0.82	

**HAC. History, Action and Capacity**			
HAC.1 Early exposure to violence-promoting, militant ideology	30	0.96***	0.92–0.98
HAC.2 Network of family and friends involved in violent extremism	30	0.78***	0.59–0.89
HAC.3 Violent criminal history	30	0.95***	0.89–0.97
HAC.4 Strategic, paramilitary and/or explosives training	30	0.94***	0.88–0.97
HAC.5 Training in extremist ideology in own country or abroad	30	0.95***	0.90–0.98
HAC.6 Organizational skills and access to funding and sources of help	30	0.51**	0.19–0.74
Mean domain HAC		0.85	

**CM. Commitment and Motivation**			
CM.1 Motivated by perceived religious obligation and/or glorification	30	0.74***	0.52–0.87
CM.2 Motivated by criminal opportunism	30	0.82***	0.66–0.91
CM.3 Motivated by camaraderie, group belonging	30	0.93***	0.86–0.97
CM.4 Motivated by moral obligation, moral superiority	30	0.82***	0.65–0.91
CM.5 Motivated by excitement and adventure	30	0.59***	0.30–0.78
CM.6 Forced participation in violent extremism	30	0.64***	0.38–0.81
CM.7 Motivated by acquisition of status	30	0.91***	0.82–0.96
CM.8 Motivated by a search for meaning and significance in life	30	0.81***	0.64–0.91
Mean domain CM		0.78	

**P. Protection and risk mitigating**			
P.1 Reinterpretation of the ideology	30	0.94***	0.88–0.97
P.2 Rejection of violence as a means to achieve goals	30	0.76***	0.55–0.88
P.3 Change in concept of the enemy	30	0.31*	0.00–0.60
P.4 Participant in programmes against violent extremism	26	0.94***	0.87–0.97
P.5 Support from the community for non-violence	30	0.89***	0.78–0.94
P.6 Support from family members, other important persons for non-violence	30	0.53**	0.22–0.74
Mean domain P		0.73	
Mean VERA-2R indicators		0.79	

ICC, Intraclass correlation coefficient and 95% CI = 95% confidence interval. The N of item P4 is 26, due to the fact that for some of the cases an suitable rating could not be assigned to the item.

*p < 0.05, **p < 0.01, ***p < 0.001.

We also found excellent interrater reliability for most of the indicators (5 of 7) within the ‘Social Context and Intention’ domain (SCI). Furthermore, good interrater reliability was demonstrated for indicator ‘SCI.3’ (ICC = 0.72), while fair interrater reliability was demonstrated for indicator ‘SCI.6’ (ICC = 0.53). The average amount of agreement over the indicators can be classified as excellent (ICC = 0.82).

The indicators representing the ‘History, Action and Capacity’ domain (HAC) all have excellent interrater reliability, with the exception of indicator ‘HAC.6’ which was found to have fair interrater reliability (ICC = 0.51). The mean ICC value of the indicators is 0.85, which indicates excellent interrater reliability.

All the indicators within the ‘Commitment and Motivation’ domain (CM) have good to excellent interrater reliability, with the exception of indicator ‘CM.5’ which was found to have fair interrater reliability (ICC = 0.59). The average amount of agreement over the indicators can be classified as excellent (ICC = 0.78).

We also found excellent interrater reliability for the majority of the indicators (4 of 6) within the ‘Protection and risk mitigating’ domain (P). Furthermore, poor interrater reliability was demonstrated for indicator ‘P.3’ (ICC = 0.31), while fair interrater reliability was demonstrated for indicator ‘P.6’ (ICC = 0.53). The mean ICC value of the indicators is 0.73, which indicates good interrater reliability.

Overall, the average amount of agreement over the VERA-2R indicators can be classified as excellent (ICC = 0.79).

### Results interrater reliability structured risk judgements

Table 2 presents the ICCs for the structured risk judgements. We found good interrater reliability for the structured risk judgements across all the domains, with the exception of the domain ‘Beliefs, Attitudes and Ideology’ (BA), which was found to have excellent interrater reliability (ICC = 0.85). With respect to the structured final risk judgement, the results reveal an excellent level of agreement between the assessors (ICC = 0.81).

**Table 2:** Interrater reliability structured risk judgements.

Structured risk judgement	*N*	ICC	95% CI
Structured risk judgement domain ‘Beliefs, Attitudes and Ideology’	30	0.85***	0.70–0.92
Structured risk judgement domain ‘Social Context and Intention’	30	0.74***	0.52–0.87
Structured risk judgement domain ‘History, Action and Capacity’	30	0.70***	0.46–0.84
Structured risk judgement domain ‘Commitment and Motivation’	30	0.74***	0.53–0.87
Structured risk judgement domain ‘Protection and risk mitigating’	30	0.74***	0.53–0.87
Structured final risk judgement	30	0.81***	0.64–0.91

ICC, Intraclass correlation coefficient and 95% CI = 95% confidence interval.

***p < 0.001.

### Interrater reliability additional indicators

Table 3 shows the kappa values for the additional indicators. The additional indicators all have good to excellent interrater reliability, with the exception of indicator ‘PH.3’, which was found to have moderate interrater reliability (κ = 0.51). Furthermore, four indicators revealed a kappa coefficient of 1, which implies perfect interrater reliability. Overall, the average amount of agreement over the additional indicators can be classified as excellent (κ = 0.85).

**Table 3:** Interrater reliability additional indicators.

Additional indicators	*N*	Kappa (κ)	95% CI
**CH. Criminal History**			
CH.1 Client of the juvenile justice system/convicted for non-violent offence(s)	30	1.00***	1.00–1.00
CH.2 Non-compliance with conditions or supervision	13	0.63*	−0.02 – 1.00
Mean domain CH		0.82	

**PH. Personal History**			
PH.1 Violence in family	30	0.90***	0.71–1.00
PH.2 Problematic upbringing and/or placed in juvenile care	30	0.80***	0.59–1.00
PH.3 Problems with school and work	30	0.51**	0.20–0.82
Mean domain PH		0.74	

**MD. Mental Disorder**			
MD.1 Personality disorder	30	0.84***	0.63–1.00
MD.2 Depressive disorder and/or suicide attempts	30	1.00***	1.00–1.00
MD.3 Psychotic and schizophrenic disorder	30	1.00***	1.00–1.00
MD.4 Autism spectrum disorder	30	1.00***	1.00–1.00
MD.5 Post-traumatic stress disorder	30	0.87***	0.62–1.00
MD.6 Substance use disorder	30	0.75***	0.49–1.00
Mean domain MD		0.91	
Mean additional indicators		0.85	

κ, kappa value and 95% CI = 95% confidence interval. The N of item CH2 is 13, due to the fact that for some of the cases an suitable rating could not be assigned to the item.

*p < 0.05, **p < 0.01, ***p < 0.001.

## Methods intra-rater reliability: assessors and cases

The assessor included in this study was a Dutch researcher (female) with a bachelor degree in psychology and a master degree in criminology. The researcher is employed by the Netherlands Institute of Forensic Psychiatry and Psychology (NIFP) of the Dutch Ministry of Justice and Security, and took part in a two-day training course to obtain an in-depth understanding of the instrument and to acquire experience in applying the VERA-2R indicators and forming structured risk judgements.

To assess the intra-rater reliability of the VERA-2R, the assessor rated a sample of terrorist cases twice, with an interval minimum of 6 months. In order to establish the minimum sample size, two *a-priori* power analyses were performed. The first *a-priori* power analysis for the intraclass correlation coefficient (ICC) estimated that 28 cases were required. We choose a minimum acceptable reliability of 0.40, since this indicates fair reliability. Furthermore, we choose an expected reliability of 0.75, since this corresponds to the interrater reliability we found for the continuous variables of the VERA-2R, and we expected that interrater reliability had a significant impact on intra-rater reliability. Furthermore, we selected a power of 0.80, a significance level of 0.05, and two repetitions per subject. The second *a-priori* power analysis for Cohen’s kappa estimated that 33 cases were required. We choose a minimum acceptable reliability of 0.40, since this indicates moderate reliability. Furthermore, we choose an expected reliability of 0.85, since this corresponds to the interrater reliability we found for the categorical variables of the VERA-2R, and we expected that interrater reliability has a significant impact on intra-rater reliability. Furthermore, we selected a proportion of outcome of 0.50, a power of 0.80 and a significance level of 0.05. Based on these power analyses, we selected 33 cases of terrorist offenders.

We assessed the 33 cases on the basis of extensive judicial files, that were provided by the Dutch Public Prosecution Service. The files included a mental health assessment, a probation report, a transcript of the verdict, a police report, a criminal record and/or information from intelligence services. The sample comprised 27 men and 6 women who were convicted of terrorist offences in the Netherlands between 2012 and 2019. The subjects’ ages ranged from 15–59 at the time of their terrorist act (*M*_age_ = 26.12, *SD* = 9.74). 84.8% of the subjects were from a migrant background, 3.0% of the subjects had no migration background, and for 12.1% the background was unknown Most of the subjects from a migrant background had parents who were born in Morocco or were born there themselves (46.4%), followed by persons with a Turkish background (17.9%). Consequently, the sample appears to be representative of the target population with respect to age, gender and migration background, since similar descriptive statistics were found in previous studies on Dutch jihadists (34–37).

### Materials: Vera-2R (See explanation above)

#### Research design

As mentioned before, our research design included a trained researcher and extensive judicial files. As a result, our research design can be said to reflect the practice of structured professional risk assessment as closely as possible. However, since, in practice VERA-2R assessments are carried out by professionals with extensive experience in carrying out individual risk assessments and preferably also include information obtained from a direct interview with the person concerned, our research design must still be defined as a research setting.

In order to ensure that the assessor did not assign different ratings on T1 and T2 as a result of relying on different sources that may have differing opinions about whether (and to what extent) the indicator characteristics are present, we decided to inform the assessor at T2 which source she had used during T1.

With respect to the interval between T1 and T2, we took into account that a long interval increases the risk of changes in the observed individual, whereas a short interval increases the risk of recall bias. Since we evaluated the cases on the basis of static judicial files, we faced no risks of changes in the observed individual. Therefore, in order to be able to minimize the risk of recall bias, we chose a long interval of 6 months.

#### Security and privacy

To ensure that there were no risks to the privacy of the subjects included in this study, we anonymized the data. Moreover, with regard to data protection, we stored the anonymized dataset in a secure digital environment, in order to protect the information against misuse, unauthorized access, disclosure and theft.

### Statistical analysis

The statistical analyses in this study were carried out using IBM SPSS Statistics for Mac Version 25.0. In order to establish the intra-rater reliability of the VERA-2R, intraclass correlation coefficient (ICC) was used (two-way mixed-effects model and absolute agreement type) (38). In line with the vision of Shrout and Fleiss (1979), the two-way mixed-effects model is chosen, as it is not reasonable to generalize the scores of one assessor to a larger population of assessors (Koo & Li, 2016). The ICC values were established for both the VERA-2R indicators and structured risk judgements. Furthermore, mean ICC values were determined by calculating the average amount of agreement over the VERA-2R indicators. Interpretation of ICCs were based on the critical values for single measures provided by Fleiss (39): ICC < 0.40 = poor, 0.40 ≤ ICC < 0.60 = fair, 0.60 ≤ ICC < 0.75 = good and ICC ≥ 0.75 = excellent.

The intra-rater reliability of the additional indicators was examined by means of Cohen’s kappa (κ). Furthermore, mean kappa values were determined by calculating the average amount of agreement over the additional indicators. The kappa values were interpreted in accordance with the guidelines outlined by Landis and Koch (40): κ ≤ 0.20 = slight, 0.20 < κ ≤ 0.40 = fair, 0.40 < κ ≤ 0.60 = moderate, 0.60 < κ ≤ 0.80 = good and 0.80 < κ ≤ 0.1.00 = excellent.

## Results

### Intra-Rater reliability VERA-2R indicators

Table 4 shows the results for the VERA-2R indicators. We found excellent intra-rater reliability for all of the indicators within the ‘Beliefs, Attitudes and Ideology’ domain (BA), with 2 indicators revealing an ICC value of 1 (BA.3 and BA.5). The mean ICC value of the indicators is 0.96, which indicates excellent intra-rater reliability.

**Table 4:** Intra-rater reliability VERA-2R.

Domain and Indicator	*N*	ICC	95% CI
**BA. Beliefs, Attitudes and Ideology**			
BA.1 Commitment to ideology that justifies violence	30	0.90***	0.80–0.95
BA.2 Perceived grievances and/or perceived injustice	30	0.98***	0.96–0.99
BA.3 Dehumanization of designated targets associated with injustice	30	1.00	1.00–1.00
BA.4 Rejection of democratic society and values	30	0.91***	0.82–0.95
BA.5 Expressed emotions in response to perceived injustice	30	1.00	1.00–1.00
BA.6 Hostility to national identity	30	0.94***	0.89–0.97
BA.7 Lack of empathy and understanding for those outside one’s own group	30	0.96***	0.92–0.98
Mean domain BA		0.96	

**SCI. Social Context and Intention**			
SCI.1 Seeker, user or developer of violent extremist materials	30	0.93***	0.86–0.97
SCI.2 Target for attack identified (person, group, location)	30	0.83***	0.68–0.91
SCI.3 Personal contact with violent extremists (informal or social context)	30	0.86***	0.73–0.93
SCI.4 Expressed intention to commit acts of violent extremism	30	0.91***	0.83–0.96
SCI.5 Expressed willingness and/or preparation to die for a cause or belief	30	0.81***	0.65–0.90
SCI.6 Planning, preparation of acts of violent extremism	30	0.94***	0.89–0.97
SCI.7 Susceptibility to influence, control or indoctrination	30	0.90***	0.79–0.95
Mean domain SCI		0.88	

**HAC. History, Action and Capacity**			
HAC.1 Early exposure to violence-promoting, militant ideology	30	1.00	1.00–1.00
HAC.2 Network of family and friends involved in violent extremism	30	0.87***	0.76–0.94
HAC.3 Violent criminal history	30	0.98***	0.95–0.99
HAC.4 Strategic, paramilitary and/or explosives training	30	0.74***	0.54–0.86
HAC.5 Training in extremist ideology in own country or abroad	30	0.95***	0.91–0.98
HAC.6 Organizational skills and access to funding and sources of help	30	0.89***	0.79–0.94
Mean domain HAC		0.91	

**CM. Commitment and Motivation**			
CM.1 Motivated by perceived religious obligation and/or glorification	30	0.81***	0.62–0.91
CM.2 Motivated by criminal opportunism	30	0.95***	0.90–0.97
CM.3 Motivated by camaraderie, group belonging	30	0.92***	0.84–0.96
CM.4 Motivated by moral obligation, moral superiority	30	0.90***	0.80–0.95
CM.5 Motivated by excitement and adventure	30	0.50**	0.21–0.72
CM.6 Forced participation in violent extremism	30	1.00	1.00–1.00
CM.7 Motivated by acquisition of status	30	0.87***	0.74–0.93
CM.8 Motivated by a search for meaning and significance in life	30	0.60***	0.33–0.79
Mean domain CM		0.82	

**P. Protection and risk mitigating**			
P.1 Reinterpretation of the ideology	30	0.80***	0.64–0.90
P.2 Rejection of violence as a means to achieve goals	30	0.82***	0.67–0.91
P.3 Change in concept of the enemy	30	0.87***	0.75–0.93
P.4 Participant in programmes against violent extremism	19	0.78***	0.51–0.91
P.5 Support from the community for non-violence	30	0.96***	0.93–0.98
P.6 Support from family members, other important persons for non-violence	30	0.57***	0.28–0.76
Mean domain P		0.80	
Mean VERA-2R indicators		0.82	

ICC = Intraclass correlation coefficient and 95% CI = 95% confidence interval. The N of item P4 is 19, due to the fact that for some of the cases an suitable rating could not be assigned to the item.

**p < 0.01, ***p < 0.001.

The indicators representing the Social Context and Intention’ domain (SCI) all have excellent intra-rater reliability, with ICC values ranging from 0.81 (indicator SCI.5) to 0.94 (indicator SCI.6). The average amount of agreement over the indicators can be classified as excellent (ICC = 0.88).

All the indicators within the ‘History, Action and Capacity’ domain (HAC) have excellent intra-rater reliability, with the exception of indicator ‘HAC.4’ which was found to have good intra-rater reliability (ICC = 0.74). Furthermore, indicator ‘HAC.1’ revealed an ICC value of 1. The mean ICC value of the indicators is 0.91, which indicates excellent intra-rater reliability.

Most of the indicators (6 of 8) within the ‘Commitment and Motivation’ domain (CM) have excellent intra-rater reliability. Furthermore, fair intra-rater reliability was demonstrated for indicator ‘CM.5’ (ICC = 0.50), while good intra-rater reliability was demonstrated for indicator ‘CM.8’ (ICC = 0.60). Indicator ‘CM.6’ moreover revealed an ICC value of 1. The average amount of agreement over the indicators can be classified as excellent (ICC = 0.82).

We also found excellent intra-rater reliability for all of the indicators within the ‘Protection and risk mitigating’ domain (P), with the exception of indicator ‘P.6’ which was found to have fair intra-rater reliability (ICC = 0.57). The mean ICC value of the indicators is 0.80, which indicates excellent intra-rater reliability.

Overall, the average amount of agreement over the VERA-2R indicators can be classified as excellent (ICC = 0.82).

### Intra-rater reliability structured risk judgements

Table 5 presents the ICCs for the structured risk judgements. We found good to excellent intra-rater reliability for the structured risk judgements across all the domains, with ICC values ranging from 0.63 (Domain BA) to 0.88 (Domain HAC). With respect to the structured final risk judgement, the results reveal an excellent level of agreement between the assessors (κ = 0.81).

**Table 5:** Intra-rater reliability structured risk judgements.

Structured risk judgement	*N*	ICC	95% CI
Structured risk judgement domain ‘Beliefs, Attitudes and Ideology’	30	0.86***	0.74–0.93
Structured risk judgement domain ‘Social Context and Intention’	30	0.64***	0.38–0.80
Structured risk judgement domain ‘History, Action and Capacity’	30	0.88***	0.77–0.94
Structured risk judgement domain ‘Commitment and Motivation’	30	0.72***	0.51–0.85
Structured risk judgement domain ‘Protection and risk mitigating’	30	0.86***	0.74–0.93
Structured final risk judgement	30	0.83***	0.68–0.91

ICC, Intraclass correlation coefficient and 95% CI = 95% confidence interval.

***p < 0.001.

### Intra-rater reliability additional indicators

The results for the additional indicators are demonstrated in Table 6. The additional indicators all have good to excellent intra-rater reliability, with the exception of indicator ‘PH.3’, which was found to have moderate intra-rater reliability (κ = 0.59). Furthermore, four indicators revealed a kappa coefficient of 1, which implies perfect intra-rater reliability. Overall, the average amount of agreement over the additional indicators can be classified as excellent (κ = 0.86).

**Table 6:** Intra-rater reliability additional indicators.

Domain and indicator	*N*	Kappa (κ)	95% CI
**CH. Criminal History**			
CH.1 Client of the juvenile justice system/convicted for non-violent offence(s)	30	0.93***	0.80–1.00
CH.2 Non-compliance with conditions or supervision	11	0.62***	−0.04–1.00
Mean domain CH		0.78	

**PH. Personal History**			
PH.1 Violence in family	30	0.74***	0.47–1.00
PH.2 Problematic upbringing and/or placed in juvenile care	30	0.94***	0.75–1.00
PH.3 Problems with school and work	30	0.59**	0.30–0.88
Mean domain PH		0.76	

**MD. Mental Disorder**			
MD.1 Personality disorder	30	1.00***	1.00–1.00
MD.2 Depressive disorder and/or suicide attempts	30	1.00***	1.00–1.00
MD.3 Psychotic and schizophrenic disorder	30	1.00***	1.00–1.00
MD.4 Autism spectrum disorder	30	1.00***	1.00–1.00
MD.5 Post-traumatic stress disorder	30	0.84***	0.53–1.00
MD.6 Substance use disorder	30	0.85***	0.65–1.00
Mean domain MD		0.95	
Mean additional indicators		0.86	

κ, kappa value and 95% CI = 95% confidence interval. The N of item CH2 is 11, due to the fact that for some of the cases an suitable rating could not be assigned to the item.

**p < 0.01, ***p < 0.001.

## Discussion

Given that risk assessments have a significant role to play in the fight against violent extremism and terrorism (6, 25), it is of importance that the risk assessment tools for violent extremism and terrorism provide reliable and valid risk assessments.

The present study investigated the reliability of the VERA-2R, focusing on the interrater- and intra-rater reliability. In accordance with our hypotheses, the results show that the reliability of the VERA-2R is good to excellent. This conclusion is first of all supported by the level of agreement over the indicators, which can be classified as excellent for both the interrater- and intra-rater reliability. These results indicate that the indicators included in the risk assessment and their encoding rules are clearly expressed (31) and can therefore be assessed in the same way by different assessors or during repeated risk assessments over time. In addition to the promising results regarding the indicators, we also found good to excellent interrater- and intra-rater reliability for the structured risk judgements, with slightly higher results for the intra-rater reliability in comparison to the interrater reliability. Although structured risk judgements have been shown to be vulnerable to subjective biases (41), these results nevertheless indicate that the way that assessors exercise their professional judgment to integrate, combine and weigh all the relevant information and data related to the indicators is stable across different assessors and during repeated risk assessments over time. This is a significant finding. First of all, because structured risk judgements serve as the basis for important decisions in the criminal justice process (19), and, as such, should be wholly independent of the observers or professional assessors (32). Secondly, it provides certainty that the VERA-2R is able to measure the risk status of an individual in the same way over time, and therefore can be used to identify changes in risk. Repeated risk assessments are relevant to re-examine the risk status over the course of time and eventually adapt the risk management, especially in the event of a change in the judicial situation, and when convicted persons return to society or during supervision by the probation service (20, 23).

The findings of the present study can be said to be in line with those found in a previous reliability study, focusing on the intra-rater reliability of the VERA, where high interrater reliability was found as well (33). However, as aforementioned, there were several limitations with Beardsley and Beech’s (33) study, namely the fact they used case vignettes, the small sample size, and their failure to include a stringent training program. Although these limitations could impact the research findings, they are nevertheless regularly reported within the field of reliability studies of structured professional risk assessment tools [e.g., (42, 43)].

Given that our research design included extensive judicial files, samples of at least 30 cases and assessors who were both trained in the use of the VERA-2R and had a bachelor and master degree in psychology and/or criminology, we were able to overcome these limitations and, in turn, recreate the practice of structured professional risk assessment as closely as possible. Therefore, the research findings provide an initial indication that the VERA-2R can produce high interrater- and intra-rater reliability in practice. Ideally, we would like to verify this further by establishing whether the same level of consistency would be found if the VERA-2R assessment was carried out by professionals with experience in carrying out individual risk assessments and also included information obtained from a direct interview with the person concerned. This research into field reliability will be challenging to investigate unequivocally, also because of the organizational and professional aspects involved in the use of the VERA-2R in practice (23). Field reliability of risk assessment instruments is often lower than reliability by highly trained raters, but they provide insights into the performance in real-world settings, exposing factors that affect reliability (44, 45). Therefore, since repeated measurements with the VERA-2R are used by professionals, the interrater reliability of the VERA-2R in practice could, and should, be established in future research.

Although in general the reliability of the VERA-2R can be classified as good to excellent, the instrument does contain risk indicators which seem to be more difficult to assess in the same way by different assessors and/or during repeated risk assessments over time. First of all, for three indicators low levels of both interrater- and intra-rater reliability were found. The interrater and intra-rater reliability of the indicator ‘Motivated by excitement and adventure’ (CM.5) may be low due to the fact that the concepts of ‘excitement’ and ‘adventure’ are not clearly defined, are missed by professionals and/or needed an even stricter search for clues in the files. As a result, it may be difficult to achieve high levels of agreement, both across different assessors and during repeated risk assessments over time. However, in order to be able to provide a clearer explanation and a well-founded recommendation for the specification and improvement of the reliability values, it might be necessary to interview professionals with extensive experience of conducting VERA-2R risk assessments. With regard to the indicator ‘Support from family members’ (P.6) one could argue that we found low interrater- and intra-rater reliability due to the fact that it is difficult to determine whether the person concerned was favourably influenced by the support. Higher levels of reliability could be achieved if the indicator focused on whether the person concerned receives prosocial or antisocial support from family members or lacks support altogether. The interrater-and intra-rater reliability of the indicator ‘Problems with school and work’ (PH.3) may be low, because the word ‘problems’ leaves room for subjective interpretation. As a result, this interpretation may differ from researcher to researcher, or from time to time. In order to increase the reliability values, one could seek to objectify the content of the indicator by replacing ‘Problems with school and work’ with ‘School dropout and work-related dismissal’. This indicator might be further specified by attitudes or behaviour like school truancy, poor work attendance, bad performance, and lack of work engagement.

In addition to this, three risk indicators were found to have high levels of intra-rater reliability, but low levels of interrater reliability. The interrater reliability of the indicator ‘Planning or preparation of acts of violent extremism’ (SCI.6), may be low due to the lack of clarity over how the indicator should be assessed if the person concerned is suspected or convicted of a crime that sought to prepare or facilitate a violent extremist or terrorist crime. This concerns specific types of crime, such as financing and incitement, where it is sometimes not clear whether these were preceded by clear preparatory acts. In order to increase the interrater reliability of this indicator, clarification is required over how the indicator should be assessed with respect to these types of crime. With regard to the indicator ‘Organizational skills and access to funding and sources of help’ (HAC.6), one could argue that the indicator encompasses too many different concepts related to the ability to plan and execute violent extremist or terrorist acts. Higher interrater reliability may thus be achieved if the indicator were to focus on the organizational skills of the person concerned, with access to funding and sources of help as constituting examples from which this could be derived. The interrater reliability of the indicator ‘Change in concept of the enemy’ (P.3) may be low due to a lack of clarity over how the indicator should be assessed if the person concerned has no enemy. Given that enemy images are closely linked to grievances about perceived injustices, higher interrater reliability could be achieved if the indicator would focus on changes in grievances.

### Limitations and recommendations

Although the present study undoubtedly provides valuable insights into the reliability of the VERA-2R, the findings should be considered in light of certain limitations. The first limitation is that, although the research design simulates the practice of structured professional risk assessment as closely as possible, it needs to be defined as a research setting. In this study, VERA-2R assessments were carried out by researchers who were trained in the use of the VERA-2R on the basis of judicial files, while, in practice, VERA-2R assessments are carried out by professionals who have extensive experience in undertaking individual risk assessments, preferably on the basis of judicial files and direct interviews with the person concerned. Since assessors must use their professional judgement to arrive at structured risk judgements (20), the use of research assessors can be criticized on the grounds of their ability to form adequate structured risk judgements. However, we partially overcame this limitation by providing a two-day training course for the researchers, in which they acquired experience in forming structured risk judgements. With regard to the exclusion of interviews, it’s important to state that the inclusion of personal interviews is not a requirement for the use of the VERA-2R. If the person concerned is absent or refuses to co-operate, VERA-2R assessments can and will be carried out without the information that would be obtained from a direct interview (20). But, very importantly, we used extensive judicial court files that also included forensic psychological and forensic psychiatric reports. A second limitation pertains to the fact that the results are based on a relative small sample size. Given that a larger sample sizes produces more reliable results with greater precision and power (46), follow-up research should be carried out to determine if the results can be replicated in a larger sample.

Taking all this into consideration, we can conclude that the present study did not only clarify the reliability of the VERA-2R, it also showed how the reliability of a structured professional risk assessment tool can be investigated with trained assessors based on extensive judicial files. As with most research, the obtained knowledge can be deepened and strengthened by carrying out further research. In addition to this, it is also necessary to strengthen the empirical foundations of the VERA-2R. Due to both the limited access to (primary) data (47) and the ethical barriers in conducting research on sensitive topics (48), the evidence-base underpinning the risk-promoting and risk-mitigating indicators for ideologically motivated violence is scant at best (14).

In order to obtain a more evidence-based professional approach to conducting violent extremism and terrorism risk assessments, the European Database of Convicted Terrorists (EDT) was developed (34). The EDT is based on judicial documents and contains personal and contextual information about convicted (and deceased) terrorists and violent extremists. By analysing this data, reliable insights could be obtained into the underlying indicators that drive individuals’ engagement, continuation or disengagement in violent extremism and terrorism. Subsequently, this would enable the validation of the VERA-2R indicators, as well as the identification of other relevant indicators vis-à-vis the risk of violent extremism and terrorism. We do realize that our extensive Dutch forensic and criminological information is rather unique for this scientific research into the VERA-2R. That might make it difficult for researchers elsewhere to replicate.

Furthermore, it is important to establish the other psychometric properties of the VERA-2R, such as the discriminative validity and divergent validity, in order to be able to develop a more reliable and valid VERA tool. These aforementioned aspects have been investigated in follow-up research. With the publication of this research, we can make more substantiated recommendations on how the VERA-2R can be improved. This could lead to more accurate risk assessments and risk management strategies.

## Data availability statement

The original contributions presented in the study are included in the article/supplementary materials, further inquiries can be directed to the corresponding author.

## Ethics statement

Ethical review and approval was not required for this study in accordance with local legislation and institutional requirements.

## Author contributions

Two former NIFP researchers participated in the study. One junior researcher wrote the research report under the supervision of ND and MK. ND wrote the article in collaboration with MK.

## Funding

This work was supported the European Commission under Grant (Call JUST-JCOO-AG-2020, Project 101007383). We want to thank them for their financial support.

## Conflict of interest

The authors declare that the research was conducted in the absence of any commercial or financial relationships that could be construed as a potential conflict of interest.

## Publisher’s note

All claims expressed in this article are solely those of the authors and do not necessarily represent those of their affiliated organizations, or those of the publisher, the editors and the reviewers. Any product that may be evaluated in this article, or claim that may be made by its manufacturer, is not guaranteed or endorsed by the publisher.
